# Evaluating Cardiac Impairment From Abnormal Respiratory Patterns: Insights From a Wireless Radar and Deep Learning Study

**DOI:** 10.1109/JTEHM.2025.3588523

**Published:** 2025-07-14

**Authors:** Chun-Chih Chiu, Wen-Te Liu, Jiunn-Horng Kang, Chun-Chao Chen, Yu-Hsuan Ho, Yu-Wen Huang, Zong-Lin Tsai, Rachel Chien, Ying-Ying Chen, Yen-Ling Chen, Nai-Wen Chang, Hung-Wen Lu, Kang-Yun Lee, Arnab Majumdar, Shu-Han Liao, Ju-Chi Liu, Cheng-Yu Tsai

**Affiliations:** Department of CardiologyTaipei Medical University-Shuang Ho Hospital 499996 New Taipei City 23561 Taiwan; School of Respiratory Therapy, College of MedicineTaipei Medical University 38032 Taipei 11031 Taiwan; Division of Pulmonary MedicineDepartment of Internal MedicineTaipei Medical University-Shuang Ho Hospital 499996 New Taipei City 23561 Taiwan; Sleep CenterTaipei Medical University-Shuang Ho Hospital 499996 New Taipei City 23561 Taiwan; Research Center of Sleep Medicine, College of MedicineTaipei Medical University 38032 Taipei 11031 Taiwan; TMU Research Center of Artificial Intelligence in Medicine and HealthTaipei Medical University 38032 Taipei 11031 Taiwan; Department of Physical Medicine and RehabilitationSchool of Medicine, College of MedicineTaipei Medical University 38032 Taipei 11031 Taiwan; Department of Physical Medicine and RehabilitationTaipei Medical University Hospital 63474 Taipei 11031 Taiwan; Graduate Institute of Nanomedicine and Medical Engineering, College of Biomedical Engineering, Taipei Medical University 38032 Taipei 11031 Taiwan; Wireless Technology and Antenna Research and Development DepartmentWistron Corporation Taipei 11469 Taiwan; Advanced Technology LaboratoryWistron Corporation Taipei 11469 Taiwan; Department of Civil and Environmental EngineeringImperial College London 4615 SW7 2AZ London U.K.; Department of Electrical and Computer EngineeringTamkang University, Tamsui 34886 New Taipei City 25137 Taiwan; School of Biomedical Engineering, College of Biomedical EngineeringTaipei Medical University 38032 Taipei 11031 Taiwan

**Keywords:** Sleep-disordered breathing, echocardiographic (2D-echo) measurements, respiratory disturbance index (RDI), periodic breathing (PB) cycle length, left ventricular ejection fraction (LVEF)

## Abstract

Objectives: Assessing the bidirectional impacts of heart function impairment and sleep-disordered breathing remains underexplored. Thus, this study analyzed respiratory patterns from a wireless radar framework to explore their associations with echocardiographic (2D-echo) measurements. Methods: Background details, 2D-echo parameters, and biochemical data were collected from patients in a cardiology ward in northern Taiwan. Their radar-based respiratory patterns from the night before and the night of the 2D-echo were obtained, averaged, and used to derive indices such as the respiratory disturbance index (RDI) and periodic breathing (PB) cycle length, representing overall respiratory patterns. Next, retrieved data were grouped based on a 50% left ventricular ejection fraction (LVEF) threshold and analyzed using mean comparisons and regression models to explore relationships. Results: Patients with an LVEF of $\le 50$% demonstrated significantly reduced total sleep time, higher RDI, and longer PB cycles compared to those with LVEF >50%. Each 1-event/h increase in the RDI reduced the LVEF by 0.22% (95% confidence interval [CI]: −0.41% to −0.03%, p <0.05), and each 1-s increase in the PB cycle length was associated with a 0.21% LVEF reduction (95% CI: −0.35% to −0.07%). Increases in RDI and PB cycle length were associated with a heightened risk of LVEF declining to $\le 50$% from >50%. Subgroup analysis revealed that the PB cycle length was associated with elevated N-terminal-prohormone-brain-natriuretic-peptide (NT-proBNP) levels. Conclusions: This study demonstrates that a wireless radar framework combined with deep learning can effectively monitor respiratory patterns that are associated with cardiac function. Its contactless nature may support continuous cardiac function assessments. Clinical Impact: This study highlights the effectiveness of a wireless radar and deep learning framework for monitoring respiratory patterns that are associated with cardiac function (e.g., LVEF), underscoring its potential for long-term cardiac and sleep-disorder management.

## I. Introduction

Heart function impairment, particularly with a left ventricular ejection fraction (LVEF) of <50%, is prevalent and often associated with multiple comorbidities, including sleep-disordered breathing (SDB) [1], [2]. The coexistence of SDB with heart function impairment can exacerbate cardiovascular complications, further impacting patient outcomes [3]. Heart function is clinically evaluated using two-dimensional echocardiography (2D-echo), which remains the gold standard. Meanwhile, the severity of SDB is typically identified through in-laboratory polysomnography (in-lab PSG). Although both approaches are non-invasive and essential for evaluation, there is currently no unified method for assessing the combined impact of heart function impairment and SDB.

Recent studies demonstrated the feasibility of monitoring the cycle length of periodic breathing (PB) to assess heart function impairment, particularly when left ventricular function is compromised [4], [5]. Researchers showed that an increase in the PB cycle length, characterized by periods of apnea followed by hyperpnea (ventilatory phases), was associated with impaired LVEF [6], [7]. This may elucidate that a detailed analysis of breathing patterns can serve as an early indicator of heart function deterioration. Additionally, monitoring respiratory patterns was proposed to screen for the risk of having SDB, such as obstructive sleep apnea (OSA), or even determine its severity [8]. Specifically, respiratory episodes such as apnea and hypopnea, which are major SDB manifestations, can be effectively identified, providing a quantifiable measure of the SDB severity. Therefore, it seems that measuring respiratory patterns may enable a combined evaluation of the impacts of heart function impairment and SDB.

To monitor respiratory patterns, researchers have proposed various approaches, such as employing sensing arrays integrated with tri-axial accelerometers and pressure sensors [9]. Other researchers indicated the possibility of using images collected through infrared cameras and motion sensor networks, then combining them with machine learning models to identify the occurrence of apnea and hypopnea [10]. However, these contact approaches or video models are still in the initial stages of development and are subject to certain limitations that may affect the accuracy of the results. Such limitations include discomfort, background illumination, and video resolution, particularly in dark sleep environments, which may further lead to poor compliance. Wireless radar techniques, such as Doppler or ultra-wideband radar, have been thoroughly investigated for their capacity to monitor respiratory patterns, thereby detecting SDB manifestations, as discussed in existing research [11], [12]. Technically, since ultra-wideband radar can represent chest wall movements in a straightforward manner, prior researchers demonstrated the feasibility of employing such wireless systems to identify apnea events during sleep in a study involving 176 participants [13]. Similarly, other researchers developed radar frameworks equipped with frequency-modulated continuous-wave radar to detect abnormal respiratory patterns, including tachypnea, bradypnea, apnea, and PB [14]. Their results revealed high accuracy in respiratory simulations during wakefulness in 20 healthy subjects. Nevertheless, in terms of efficacy and cost, these suggested techniques may be relatively expensive and require significant hardware for signal transmission and reception, compared to continuous-wave radar [15]. In other words, under circumstances involving short distances (e.g., from the headboard to the bed), continuous-wave radar may offer a more-viable option for practical use due to its cost-efficiency and satisfactory precision in capturing chest wall movements to measure respiratory patterns. Researchers employed a continuous-wave radar framework to predict the respiratory disturbance index (RDI) and compared it to the apnea-hypopnea index (AHI) derived from PSG, which demonstrated high significant correlations [16]. However, there is insufficient evidence to validate the use of continuous-wave radar for monitoring specific respiratory patterns, particularly the identification of the PB cycle length during sleep. Thus, further study is required to verify and clarify the feasibility of employing such a wireless radar framework.

In this retrospective study, we investigated the clinical applicability and translational potential of using a contactless radar system for cardiac dysfunction assessments through a respiratory pattern analysis. We explored the feasibility of employing respiratory pattern indices measured by a wireless, continuous-wave radar framework to assess the risk of heart function impairment (LVEF of $\le 50$%). The hypothesis was that radar-based respiratory pattern indices, such as the PB cycle length and RDI, could be effectively analyzed with hybrid deep learning models to assess associations with 2D-echo measurements and other cardiac-related biomarkers. This could facilitate the evaluation of heart function. The present findings highlight the potential of the proposed wireless radar framework as a noninvasive tool for assessing cardiac function and SDB severity, offering a clinically applicable approach for both hospital-based and home-based monitoring settings.

## II. Materials and Methods

### A. Eligible Patient List Determination

This study retrospectively collected a patient list from the cardiology ward at a Hospital in New Taipei City, Taiwan, from July 2023 to March 2024. The initial inclusion criteria required that patients had undergone 2D-echo and stayed in a ward while being monitored by a wireless radar system, to continuously observe respiratory patterns throughout their hospital admission. Exclusion criteria included: (1) individuals under 20 years of age due to ethical considerations, (2) regular users of psychotropic or hypnotic medications that could alter respiratory patterns, (3) not having had a 2D-echo examination during hospitalization, (4) currently undergoing cancer treatment, and (5) being pregnant. Background details, personal habits, comorbidities, biochemistry data, and 2D-echo measurements of all eligible patients from the derived lists were collected during the admission period for further analysis.

### B. Background, Echocardiographic Details, and Biochemical Data

This study initially obtained medical records of eligible patients and retrospectively retrieved relevant data from the electronic medical record system. Detailed background information was collected, including age, gender, body-mass index (BMI), smoking status, and diagnosis at admission. Their background of underlying comorbidities was determined, and the Charlson comorbidity index (CCI) was subsequently obtained [17]. The date and measurements from 2D-echo were extracted, including the LVEF, left ventricular end-diastolic diameter (LVEDD), and left ventricular end-systolic diameter (LVESD), as reported in 2D-echo records. Specifically, the 2D-echo examinations were performed by cardiologists using the Cardiac Ultrasound System (Philips iE33, Philips, Andover, MA, USA). With the patient in a supine position, the cardiologist positioned the transducer on the anterior thorax and utilized M-mode to capture sequential cross-sectional images of the ventricle throughout the cardiac cycle. Next, Simpson’s method was applied for 3D reconstruction to derive ventricular volumes from echocardiographic images across multiple planes. A minimum of three consecutive heartbeats were recorded while the patient was at rest, and the relevant measurements were averaged. Lastly, biochemical and hematological tests, performed within 1–3 days of the 2D-echo, were also collected. Notably, this study also determined some biomarkers related to the cardiac function status, such as troponin I and N-terminal prohormone of the brain natriuretic peptide (NT-proBNP), to investigate associations between respiratory patterns and biomarker levels. However, these specific biomarker concentrations were not routinely measured and were instead analyzed as part of subgroup analyses in the present study.

### C. Wireless Radar Framework and Measured Parameters

**FIGURE 1.**
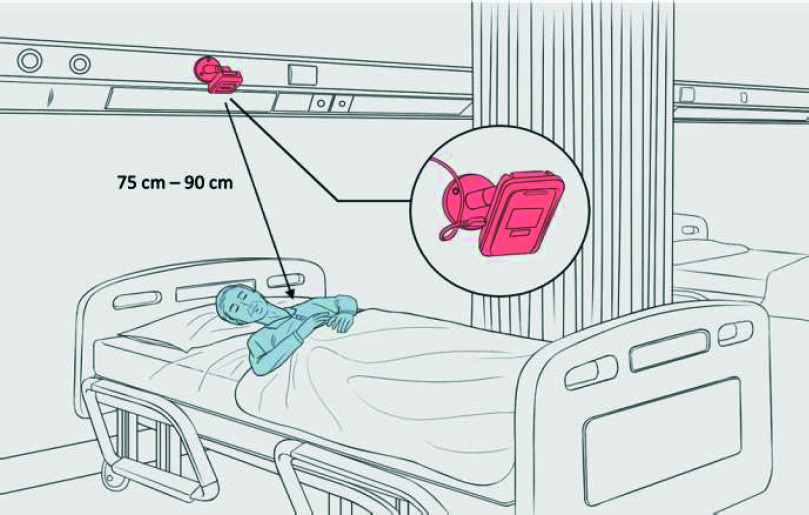
The wireless radar device was positioned 75–90 cm in front of the bed to achieve optimal respiratory waveform signal detection.

**FIGURE 2.**
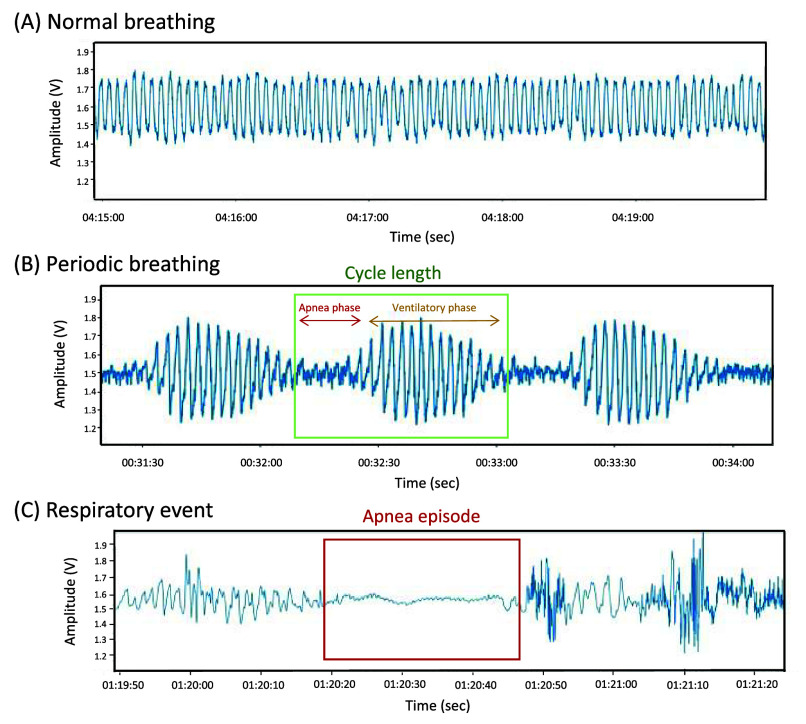
Respiratory Signal Waveforms Obtained via the Wireless Radar System. Continuous-wave signals, labeled as detected respiratory waveforms, were transformed into amplitude representations(voltage-based) to display respiratory patterns such as normal breathing (Fig 2A), periodic breathing (Fig 2B), and respiratory events (Fig 2C).

The wireless radar system installed in the cardiology ward was developed by a Medical Technology company in New Taipei City, Taiwan. This system was certified for safety in the motion detection wireless category by the Taiwan National Communications Commission (CCAF19LP2510T5) and was approved as a medical device intended for non-contact respiratory pattern monitoring by the Taiwan Ministry of Health and Welfare (license no. 007955). As to the specifications and embedded units, the radar framework consists of a sensor box equipped with radio frequency modules that capture continuous-wave signals for monitoring (**Supplementary Table S1**). The sensor box operates at a 24-GHz frequency, emitting continuous waves with a wavelength of approximately 12.5 mm, which is ideal for tracking chest wall movements. Typically, respiratory movements result in chest displacements ranging from 1 to 12 mm, while cardiac activities cause variations of 0.1–0.5 mm. Positioned between 1 and 1.5 m from a subject, this 24-GHz radar frequency is highly effective for clinically measuring chest wall movements during the respiratory cycle. Technically, the radar sensor transmits radio frequency signals directed at a participant reclining on a ward bed and then records the reflected signals to determine respiratory signals. The signals were subsequently processed using hybrid deep neural decision tree models, which integrate conventional machine learning and deep learning features. The model architecture is illustrated in Supplementary Figure S1. The hyperparameters for these models were tuned following procedures established in prior relevant studies [16], [18]. After these models were optimized, they were trained and validated on a dataset comprising 29,496 30-s epochs. Using these hybrid models, the radar framework was able to discern times of in-bed and off-bed periods and further estimates wakefulness and various sleep stages, specifically rapid eye movement (REM) and non-REM (NREM). The wireless radar device was installed at the head of the bed in the cardiology ward without the need for participants to wear additional sensors (Figure. 1). Two primary respiratory pattern indices were subsequently obtained: the PB cycle length and the RDI. Technically, the measured respiratory signals were analyzed using entropy calculations to detect distinct breathing patterns, such as peak-to-peak and valley-to-valley intervals and peak amplitudes (Figure. 2A). First, to measure the PB cycle length, breathing patterns that met the definition of periodic breathing—a crescendo-decrescendo pattern of hyperventilation between central apneas or hypopneas (for at least three consecutive cycles)—were identified [19]. The PB cycle length was then calculated by determining the duration of paired apneic and ventilatory phases (Figure. 2B). To determine the RDI, the identified breathing features were input into established hybrid models, specifically deep neural decision trees, to predict the occurrence of apnea or hypopnea events (Figure. 2C). Following this, the aggregated predicted number of respiratory episodes was calculated and divided by the estimated total sleep time to determine the RDI. Radar-based respiratory pattern indices derived from the previous night and the night of the 2D-echo were averaged to represent overall respiratory patterns for further analysis.

### D. Statistical Examination

The present study classified the parameters of retrieved individuals by setting the cut-point at an LVEF of 50%. The open-source statistics library Scikit-learn (vers. 0.21.2), implemented in Python, was used to analyze both continuous and categorical data, generating statistical outcomes, including descriptive and regression results [20]. First, descriptive statistics for baseline characteristics, 2D-echo measurements, and biochemical levels are presented as means and standard deviations (SDs), and comparisons of means were performed. For associations of the determined radar-based respiratory pattern indices with 2D-echo measurements and biochemical levels, multivariable linear regression models adjusted for age, sex, and BMI were conducted. To investigate the odds ratios (ORs) with 95% confidence intervals (CIs) for radar-based respiratory pattern indices between groups with a higher or lower LVEF than 50%, multivariable logistic regression models, subject to adjustment for age, sex, and BMI, were employed.

## III. Results

### A. Baseline Characteristics, Echocardiographic Measures, and Biochemical Parameters

There were 72 patients with comprehensive retrospective clinical data included in this study, with 47 in the group with an LVEF of >50% and 25 in the group with an LVEF of $\le 50$%. Their baseline characteristics, primary admission diagnoses, smoking status, and 2D-echo measures are reported in Table 1. The mean ages were 73.45 (SD =13.46) and 70.61 (SD =13.71) years, respectively. The majority of the group with an LVEF of $\le 50$% were male. Distributions of primary admission diagnoses and CCI between the two groups did not statistically significantly differ. Most patients in both groups did not smoke (44% vs. 61.7%). Regarding 2D-echo measures, the group with an LVEF of $\le 50$% had a mean LVEF of 36.04% (SD =9.52%), while the group with an LVEF of >50% had a mean LVEF of 65.41% (SD =9.41%). For the biochemical and hematologic tests, no measures showed statistically significant differences between the groups (Table 2). In the subgroup analysis, which included 17 patients in the group with an LVEF of >50% and 16 patients in the group with an LVEF of $\le 50$%, those with an LVEF of ≤ 50% demonstrated significantly higher mean NT-proBNP levels at 7475.79 (SD =7318.69)pg/mL compared to the group with an LVEF of >50%, which had a mean of 6737.23 (SD =15,529.39) pg/mL.

**TABLE 1:** Comparisons of Characteristic Variables Between Individuals Grouped by a 50% Left Ventricular Ejection Fraction (LVEF) Threshold

Variable	LVEF >50% ( $N =47$)	LVEF ≤ 50% ( $N =25$)	*p* value
Age (years) a	73.45 ± 14.38	70.61 ± 13.71	0.26
Gender (male/female)^b^	25 / 22	18 / 7	0.14
Body-mass index (kg/m ${}^{2}) ^{a}$	24.83 ± 4.52	25.82 ± 4.02	0.28
Charlson comorbidity index (score) a	4.13 ± 1.87	3.88 ± 1.69	0.59
Primary admission diagnosis, *n* (%)			0.87
Heart failure	12 (25.53%)	7 (28%)	
Hypertension	10 (21.27%)	3 (12%)	
Arrhythmia	8 (12.02%)	4 (16%)	
Myocardial infarction	9 (19.14%)	8 (32%)	
Coronary heart disease	4 (14.81%)	2 (8%)	
Heart valve disease	2 (4.25%)	1 (4%)	
Septic shock	2 (4.25%)	0 (0%)	
Smoking status, *n* (%)			0.22
Current smoker	12 (25.53%)	7 (28%)	
Ex-smoker	6 (12.76%)	7 (28%)	
Non-smoker	29 (61.70%)	11 (44%)	
Echocardiographic details			
LVEF (%) a	65.41 ± 9.41	36.04 ± 9.52	<0.01
LVEDD (mm)^a^	45.79 ± 8.14	56.52 ± 10.15	<0.01
LVESD (mm)^a^	28.79 ± 7.17	46.48 ± 10.23	<0.01

Abbreviations: LVEDD, left ventricular end-diastolic diameter; LVESD, left ventricular end-systolic diameter. Data are expressed as the mean ± standard deviation.

^a^ Differences between groups were assessed using the Mann-Whitney U-test.

^b^ Differences between groups were assessed using the Fisher’s exact test

**TABLE 2:** Comparisons of Biochemical Parameters Between Individuals Grouped by a 50% Left Ventricular Ejection Fraction (LVEF) Threshold

Variable	LVEF >50% ( $N =47$)	LVEF ≤ 50% ( $N =25$)	*p* value
Creatinine (mg/dL)	1.76 ± 1.88	1.88 ± 1.93	0.21
eGFR (mL/min/1.73 m ${}^{2})^{}$	64.59 ± 32.29	62.71 ± 32.67	0.99
WBCs ( $10^{\mathbf {3}}$/ $\mu $L)	8.34 ± 2.84	9.31 ± 3.99	0.33
RBCs ( $10^{\mathbf {6}}$/ $\mu $L)	25.34 ± 146.49	4.41 ± 1.07	0.06
Hemoglobin (g/dL)	11.74 ± 2.54	12.79 ± 3.64	0.09
Platelets ( $10^{\mathbf {3}}$/ $\mu $L)	195.39 ± 72.57	219.32 ± 59.61	0.05
HCT (%)	34.34 ± 6.98	38.11 ± 9.26	0.06
Subgroup analysies#			
Troponin I (pg/mL)	2955.82 ± 5538.13	8120.6 ± 23074.41	0.71
NT-proBNP (pg/mL)	6737.23 ± 15529.39	7475.79 ± 7318.69	0.04

Abbreviations: eGFR, estimated glomerular filtration rate; WBCs, white blood cells; RBCs, red blood cells; HCT, hematocrit; NT-proBNP, N-terminal prohormone of the brain natriuretic peptide.

Data are expressed as the mean ± standard deviation.

Differences between groups were assessed using the Mann-Whitney U-test.

^#^ Subgroup analysis: Data from 17 patients in the group with an LVEF of >50% and 16 patients in the group with an LVEF of ≤ 50% were analyzed.

### B. Comparisons of Radar-Based Sleep Parameters and Respiratory Pattern Indices

Supplementary Table S2 provides a comparative overview of the proposed continuous-wave radar system and other commonly used respiratory monitoring approaches, including frequency-modulated continuous-wave radar, video-based systems, and wearable sensors. This comparison highlights key parameters such as system costs, detection capabilities, monitoring durations, and setup complexity. Notably, the proposed continuous-wave radar system demonstrates cost-effectiveness due to its simple installation and suitability for long-term monitoring at a relatively low cost.

Comparisons of mean of radar-based measures, including sleep parameters and respiratory pattern indices, are presented in Table 3. Compared to patients in the group with an LVEF of >50%, those with an LVEF of ≤ 50% demonstrated significantly lower means in total sleep time (p =0.01) and sleep efficiency (p <0.01). For radar-based respiratory pattern indices, patients with an LVEF of ≤ 50% showed significantly higher means in the RDI at 44.13 (SD =20.76) events/h and the extended PB cycle length at 33.21 (SD =29.85) s compared to the group with an LVEF of >50%, which had a mean RDI of 31.91 (SD =20.74) events/h and a mean PB cycle length of 14.72 (SD =24.19) s.

**TABLE 3:** Comparisons of Radar-Based Parameters Between Individuals Grouped by a 50% Left Ventricular Ejection Fraction (LVEF) Threshold

Variable	LVEF >50% ( $N =47$)	LVEF ≤ 50% ( $N =25$)	*p* value
Radar-based sleep parameters			
TST (min)	283.74 ± 101.13	213.88 ± 91.39	0.01
Sleep efficiency (%)	49.47 ± 17.35	36.64 ± 15.25	<0.01
RDI (events/h)	31.91 ± 20.74	44.13 ± 20.76	0.02
PB cycle length (s)	14.72 ± 24.19	33.21 ± 29.85	0.01

Abbreviations: TST, total sleep time; RDI, respiratory dysfunction index; PB, periodic breathing.

Differences between groups were assessed using the Mann-Whitney U-test.

Data are expressed as the mean ± standard deviation.

The dataset comprised 15,264 apnea and hypopnea events, alongside 14,232 segments of normal data with no respiratory events. As presented in Supplementary Table S3, the established hybrid deep learning model achieved an overall classification accuracy exceeding 75%, demonstrating its effectiveness in identifying respiratory events.

### C. Associations of Radar-Based Respiratory Pattern Indices with Echocardiographic Measures and Biochemical Parameters

Associations of radar-based respiratory pattern indices with 2D-echo and biochemical parameters, as determined by multivariable linear regression models, are documented in Table 4. After adjusting for age, gender, and BMI, an increase of 1 event/h in the RDI was significantly associated with a decrease of 0.22% in the LVEF (95% CI: −0.41% to −0.03%, p <0.05) and an increase of 0.14 mm in the LVESD (95% CI: 0.01 to 0.27 mm, p <0.05). Similarly, an increase of 1 s in the PB cycle length was significantly associated with a decrease of 0.21% in the LVEF (95% CI: −0.35% to −0.07%), an increase of 0.11 mm in the LVEDD (95% CI: 0.01 to 0.19 mm, p <0.05), and an increase of 0.16 mm in the LVESD (95% CI: 0.06 to 0.25 mm, p <0.01) after adjusting for age, gender, and BMI. In the subgroup analysis (N =33), an increase of 1 s in the PB cycle length was significantly associated with an increased level of 158.49 pg/mL in NT-proBNP (95% CI: 16.31 to 300.67 pg/mL, p <0.05), after adjusting for age, gender, and BMI.

**TABLE 4:** Associations of Radar-Based Parameters With Echocardiographic and Biochemical Details

Variable	$\beta $ coefficient (95% CI)	
	RDI (events/h)	PB cycle length (s)
Biochemical details		
Creatinine (mg/dL)	0.01 (−0.01 to 0.04)	0.01 (-0.01 to 0.02)
eGFR (mL/min/1.73m*^2^*)	−0.21 (-0.57 to 0.14)	−0.07 (-0.34 to 0.2)
WBCs (10^3^/ $\mu $L)	0.01 (-0.03 to 0.05)	0.002 (-0.03 to 0.03)
RBCs (10^6^/ $\mu $L)	−0.23 (-1.64 to 1.18)	−0.3 (-1.37 to 0.77)
Hemoglobin (g/dL)	−0.01 (-0.04 to 0.02)	0.002 (-0.02 to 0.03)
Platelets (10^3^/ $\mu $L)	0.01 (-0.81 to 0.83)	0.42 (-0.2 to 1.03)
HCT (%)	−0.01 (-0.09 to 0.07)	0.01 (-0.05 to 0.07)
Subgroup analysis#		
Troponin I (pg/mL)	−62.13 (-361.08 to 236.83)	−1.97 (-210.72 to 206.78)
NT-proBNP (pg/mL)	66.65 (-153.2 to 286.49)	158.49 (16.31 to 300.67) *
Echocardiographic details		
LVEF (%)	−0.22 (-0.41 to -0.03) *	−0.21 (-0.35 to -0.07) *
LVEDD (mm)	0.07 (-0.05 to 0.19)	0.11 (0.01 to 0.19) *
LVESD (mm)	0.14 (0.01 to 0.27) *	0.16 (0.06 to 0.25) **

Abbreviations: eGFR, estimated glomerular filtration rate; WBCs, white blood cells; RBCs, red blood cells; HCT, hematocrit; LVEF, left ventricular ejection fraction; LVEDD, left ventricular end-diastolic diameter; LVESD, left ventricular end-systolic diameter; NT-proBNP, N-terminal prohormone brain natriuretic peptide; RDI, respiratory dysfunction index; PB, periodic breathing.

^#^ Subgroup analysis: Data from 17 patients in the group with an LVEF of >50% and 16 patients in the group with an LVEF of ≤ 50% were analyzed. Multivariable linear regression models were adjusted for age, gender, and body-mass index. $\ast p < 0.05$; $\ast \ast p < 0.01$.

### D. Associations Between Radar-Based Respiratory Pattern Indices and Heart Function Impairment

Associations of radar-based respiratory pattern indices among patients with an LVEF of >50% and those with an LVEF of ≤ 50%, assessed using logistic regression models, are presented in Table 5. In the crude models, an increase of 1 event/h in the RDI and an increase of 1 s in the PB cycle length were significantly associated with a 1.03-fold (95% CI: 1.01 to 1.05, p <0.05) and a 1.02-fold (95% CI: 1.01 to 1.04, p <0.01) elevated OR of impaired LVEF from >50% to ≤ 50%, respectively. In the adjusted model (after adjusting for age, gender, and BMI), an increase of 1 event/h in the RDI and an increase of 1 s in the PB cycle length similarly were significantly associated with a 1.04-fold (95% CI: 1.01 to 1.07, p <0.05) and a 1.03-fold (95% CI: 1.01 to 1.05, p <0.05) elevated OR of impaired LVEF from >50% to ≤ 50%, respectively.

**TABLE 5:** Associations (Odds Ratios, ORs) of Radar-Based Sleep Parameters Between Individuals Grouped by a 50% Left Ventricular Ejection Fraction (LVEF) Threshold

Variable	Crude OR (95% CI) a	Adjusted OR (95% CI) b
RDI (events/h)	1.03 (1.01 to 1.05) *	1.04 (1.01 to 1.07) *
PB cycle length (s)	1.02 (1.01 to 1.04) **	1.03 (1.01 to 1.05) *

Abbreviations: RDI, respiratory dysfunction index; PB, periodic breathing; CI, confidence interval.

^a^ Crude OR: Simple logistic regression models.

^b^ Adjusted OR: Multivariable logistic regression models adjusted for age, gender, body-mass index.

^*^ $p < 0.05$;

^**^ $p < 0.01$.

## IV. Discussion

In this study, we aimed to advance the application of monitoring approaches under the complex interplay between respiratory abnormalities during sleep and cardiac dysfunction, specifically in terms of heart function impairment and SDB. By leveraging a routinely used wireless radar framework coupled with deep learning techniques, recorded respiratory patterns and derived indices were obtained during sleep. First, means of these indices between patients by setting the cut-point at an LVEF of 50% were compared. Subsequently, associations of these indices with the obtained 2D-echo measures and subgroup analyses of cardiac-related biomarkers were investigated. The present findings indicated that increases in the RDI and extended PB cycle length may serve as indicators of deteriorating cardiac function, with particularly significant evidence of these associations in the LVEF. In the subgroup analysis, an extended PB cycle length was significantly associated with elevated NT-proBNP levels. Additionally, both the derived RDI and PB cycle length were significantly associated with a reduced LVEF (≤ 50%).

Regarding differences in biochemical parameters between the two groups, NT-proBNP levels were significantly higher in patients with an LVEF of ≤ 50% compared to those with an LVEF of >50%, although these comparisons were subjected to subgroup analysis due to practical limitations. This observation may be attributed to increased cardiac wall stress and diastolic dysfunction, which can cause an elevation of NT-proBNP levels even when systolic function appears normal. Clinically, elevated NT-proBNP is suggested to reflect subtle changes in cardiac pressure and volume [21]. In terms of radar-based parameters, patients with an LVEF of ≤ 50% exhibited significantly decreased total sleep time and sleep efficiency, alongside an increased RDI and prolonged PB cycle length, compared to those with an LVEF of >50%. Diastolic dysfunction and pulmonary congestion may explain these observations. Specifically, diastolic dysfunction often leads to increased pulmonary pressure and congestion, which can trigger frequent arousals and disrupt total sleep time [22], thereby reducing sleep efficiency [23]. Additionally, impaired cardiac output and blood flow in patients with an LVEF of ≤ 50%, associated with reduced pumping efficiency, may decrease cardiac output and impair blood flow to the cerebral respiratory centers. This impairment can hinder responses in detecting blood gas changes, contributing to the cycle of apnea and hyperventilation observed with an increased RDI or prolonged PB cycle length [24]. Dysregulation of the autonomic nervous system, such as increased sympathetic activity, in patients with impaired heart function can affect respiratory control and stability, promoting periodic breathing patterns [25]. Additionally, patients with impaired heart function often exhibit enhanced chemoreceptor sensitivity to changes in blood carbon dioxide levels. This heightened response leads to overcompensation, resulting in frequent episodes of apnea or hypopnea and prolonged PB cycle lengths [26]. Altogether, elevated levels of NT-proBNP, reduced sleep efficiency, decreased total sleep times, frequent respiratory events, and an extended PB breathing cycle in patients with an LVEF of ≤ 50% were likely consequences of impaired cardiac function, prolonged circulation times, and autonomic dysregulation, which collectively disrupt respiratory feedback mechanisms.

Associations of radar-based respiratory pattern indices (e.g., RDI and PB cycle length) with alterations in NT-proBNP levels and 2D-echo measures (e.g., LVEF and LVESD) were determined. Significant ORs associated with the risk of impaired heart function, particularly in cases where the LVEF declined from >50% to ≤ 50%, were also observed. A plausible interpretation of these significant associations may involve the bidirectional relationship between impaired cardiac function and respiratory control mechanisms. Specifically, as mentioned above, diastolic dysfunction and elevated cardiac stress may exacerbate disturbances in sleep breathing patterns, increasing the frequency of respiratory events or prolonging the PB cycle length. In another aspect, recurrent nocturnal respiratory episodes may further compromise cardiac function, demonstrating the intricate interplay between cardiovascular and respiratory pathophysiologies. Specifically, recurrent apnea and hypopnea events cause intermittent hypoxia, triggering oxidative stress, systemic inflammation, and increased sympathetic nervous system activity. This cascade impairs endothelial function, elevates heart rate and blood pressure, and heightens cardiac workload, contributing to cardiac stress and potentially worsening heart failure through cardiac remodeling [27], [28]. Additionally, the hemodynamic effects of SDB, such as surges in intrathoracic pressure and decreased impedance due to airway obstruction, result in significant inspiratory efforts against a partially or fully closed airway, generating large negative intrathoracic pressures [29]. These pressures draw more blood into the thoracic cavity, increasing fluid volume, impairing left ventricular function, elevating cardiac wall stress, and stimulating the release of NT-proBNP, a biomarker of heart failure [30]. Given that impaired cardiac function can impact the PB cycle length, it is reasonable to consider that the PB cycle length might also serve as a quantitative indicator of cardiac function deterioration [31]. The observed prolongation of the PB cycle length in patients with a reduced ejection fraction suggested a potential reciprocal concept, where an alteration in the PB cycle length may reflect the severity of cardiac dysfunction [32]. Therefore, monitoring the PB cycle length may provide valuable insights into the progression of heart failure, offering a non-invasive method to assess and track exacerbations in cardiac performance.

Regarding clinical applications, the proposed radar framework demonstrates potential for continuous, contactless monitoring of respiratory patterns, which may serve as indicators associated with cardiac function, without causing patient discomfort. Similarly, previous research revealed the feasibility of a contactless RF-based (60–64 GHz) sensing system for long-term heart rate variability monitoring, achieving lower estimation errors and improved accuracy compared to conventional approaches [33]. In terms of the characteristics of the proposed radar framework, compared to other radar mechanisms, such as the frequency-modulated continuous-wave system, this study employed 24-GHz continuous-wave methods to monitor breathing patterns of subjects. Although advanced radar systems, such as frequency-modulated continuous-wave, can provide relatively detailed velocity and range measurements for physiological movements (e.g., subtle chest wall movements), such approaches require more computational resources and corresponding financial commitments, which may limit their further clinical generalization [34]. Furthermore, this study primarily focused on respiratory actions during sleep, which typically result in chest displacements ranging 1–12 mm, while heart-related activities produce shifts of 0.1–0.5 mm. Utilizing continuous-wave radar, positioned 1–1.5 m away from the thoracic area, may provide precise resolution at a reasonable cost for monitoring such physiological activities, particularly breathing patterns during sleep [35]. Regarding implementation, the proposed continuous-wave radar system was designed with an emphasis on affordability, encompassing both hardware and system deployment (**Supplementary Table S2**). In comparison, commercially available frequency-modulated continuous-wave radar development kits typically require substantially greater resources for hardware alone, excluding additional software or processing components. Additionally, it is crucial to balance frequency with sensitivity when evaluating radar systems. Higher frequencies typically offer enhanced resolution and sensitivity, which are useful for detecting nuanced movements and reflections due to their shorter wavelengths. However, their penetration capabilities are limited, as high-frequency signals tend to be obstructed and reflected more easily, losing effectiveness with distance and restricting their utility in long-range detection scenarios. Conversely, lower frequencies better penetrate through objects and clothing but at the cost of reduced resolution and sensitivity. The 24-GHz continuous-wave radar, with a wavelength of approximately 12.5 mm, was found to be adequate for capturing typical physiological movements associated with respiratory and cardiac activities [36], [37]. Compared to other types of radar (**Supplementary Table S2**), the 24-GHz continuous-wave radar employed in this study appears to be a more-suitable option for clinical measurement of breathing patterns. This is particularly effective when subjects are positioned at an optimal distance from the measurement device which is directly in front of the bed.

There are several strengths of and significant contributions by the current study. First, the proposed radar framework, enhanced with deep learning techniques, effectively captured and analyzed respiratory patterns during sleep, providing a reliable method to evaluate cardiac function using derived indices. Notably, the study determined significant associations between radar-derived indices (RDI and PB cycle length) and 2D-echo measurements, confirming the potential of this non-invasive technology for cardiac function assessments. Specifically, an increased RDI and extended PB cycle length were identified as dependable markers associated with reduced cardiac function, suggesting potential utility for identifying individuals with an impaired LVEF. Additionally, in subgroup analyses, an extended PB cycle length was associated with elevated NT-proBNP levels, affirming its predictive value for cardiac dysfunction. Additionally, alterations in the RDI and PB cycle length were significantly associated with an elevated risk of impaired heart function from >50% to ≤ 50% LVEF, highlighting the possibility of again emphasizing their reliable cardiac function indicators. Collectively, these outcomes underscore the significant contributions in advancing our understanding of the complex interplay between respiratory and cardiac dysfunctions during sleep, and the efficacy of integrating radar technology with deep learning models for precise clinical assessments. Furthermore, the proposed radar devices can be easily installed in a home environment and do not require continuous on-site monitoring. This makes them an ideal option for home-based, long-term monitoring, as well as for the thorough analysis of comprehensive data to evaluate SDB evaluations and cardiac function management in clinical settings.

While this study offered valuable insights into the associations between respiratory patterns and heart function impairments, it also has several limitations that should be acknowledged and further addressed. First, the retrospective nature may have introduced biases related to data collection and patient selection. Data were collected solely from patients admitted to a single cardiology ward and lacked longitudinal follow-up, potentially limiting the generalizability of the results to other clinical settings. Additionally, the sample size of 72 patients may be too small to effectively generalize the findings to a broader population. The limited diversity of the sample in terms of age, gender, and ethnic backgrounds could also impact the applicability of the results across different patient groups. The present study excluded patients who regularly employed psychotropic or hypnotic medications to avoid alterations in respiratory patterns. Nonetheless, additional elements like smoking habits, unique craniofacial features, oral hygiene, and the anatomical structure of upper airway obstructions may also influence breathing patterns (e.g., buccal respiration). These factors ought to be taken into account when implementing the proposed system [28], [38]. Next, the continuous-wave radar system demonstrated a robust performance in identifying respiratory events, even when data were collected across various sleep positions in a natural environment. Although the system can distinguish between multiple individuals and differentiate them from non-human objects on the bed, its performance may still be affected by environmental motion. In particular, both voluntary and involuntary patient movements, as well as other dynamic elements within the radar’s field of view, can reduce the signal quality and the reliability of extracted features. While basic signal-filtering techniques were applied during preprocessing to mitigate such artifacts, more-advanced methods—such as motion compensation or data segmentation—were not employed in this study. Incorporating such strategies in future work may further enhance the robustness and clinical applicability of the proposed framework. Although the radar system effectively captured chest signals indicative of SDB, its primary function was limited to identifying respiratory events (i.e., apnea and hypopnea events) and the occurrence of PB. However, it did not possess the capability to distinguish between central and obstructive sleep apnea types. For the combined software approaches, the present hybrid models, developed from data across multiple sleep positions, did not thoroughly assess how these positions affect respiration pattern frequencies [39]. Moreover, the lack of oximetric data in our study, while not critical, restricted our ability to evaluate oxygen desaturation associated with SDB. Future integration of oximetry with the radar system could provide a more-comprehensive analysis of SDB severity, correlating respiratory events with oxygen levels to enhance reliability without notably increasing costs [40], [41]. In terms of cardiac biomarkers, the use of NT-proBNP levels and echo-derived indices for assessing cardiac function was constrained by the limited number of cases, which may hinder a nuanced understanding of relationships between cardiac health and derived respiratory pattern indices. Additionally, although extraction of Doppler cardiogram (DCG) signals from the radar system is technically feasible and under development, it was not implemented in this study. Incorporating DCG features may offer additional cardiac-specific information beyond respiration-induced movement, potentially enhancing the clinical utility of the proposed framework. Future enhancements to the framework, such as capturing whole-body signals and incorporating DCG-based features, may enable a more-comprehensive evaluation of both sleep disorders and cardiac impairment. To address these limitations and strengthen the robustness of the findings, future prospective multicenter studies should involve larger and more-diverse cohorts. Real-world implementation studies and continuous monitoring trials are essential to further validate the performance, generalizability, and clinical utility of the proposed radar framework in practical healthcare settings.

## V. Abbreviations

BMI, body-mass index; CCI, Charlson comorbidity index; CI, confidence interval; DCG, Doppler cardiogram; In-lab PSG, in-laboratory polysomnography; LVEDD, left ventricular end-diastolic diameter; LVEF, left ventricular ejection fraction; LVESD, left ventricular end-systolic diameter; NREM, non-rapid eye movement; NT-proBNP, N-terminal prohormone brain natriuretic peptide; OR, odds ratio; PB, periodic breathing; RDI, respiratory disturbance index; REM, rapid eye movement; SD, standard deviation; SDB, sleep-disordered breathing; 2D-echo, echocardiography.

## VI. Conclusion

Given the limited development in assessing the combined impacts of heart function impairment and SDB, this study retrospectively collected breathing patterns from a wireless radar framework and analyzed the respiratory pattern indices during sleep to investigate their associations with 2D-echo measurements and cardiac-related biomarkers. Relevant data (e.g., background, biochemical parameters, respiratory patterns from the radar framework, and 2D-echo measurements) were obtained and further analyzed from 72 patients admitted to a cardiology ward. As to study outcomes, significant associations of radar-based respiratory pattern indices (i.e., an increased RDI and extended PB cycle length) with a decline in the LVEF and an elevated LVESD were observed. For the subgroup analysis, an increased PB cycle length was also significantly associated with elevated NT-proBNP levels. Additionally, both an increased RDI and extended PB cycle length were significantly associated with an elevated risk of impaired LVEF declining from >50% to ≤ 50%. Collectively, this study sheds light on the utility of the proposed wireless radar framework combined with deep learning methods for monitoring respiratory breathing patterns and thereby evaluating heart function in advancing our understanding of their complex interplay. Moreover, the derived RDI and PB cycle length, as calculated by the proposed system, may be capable of serving as indicators for assessing the risk of impaired LVEF severity, particularly in cases declining from >50% to ≤ 50%. The contactless nature and easy home installation enable continuous, long-term tracking without on-site monitoring, making it ideal for home-based care and providing valuable insights for personalized cardiac and respiratory management. Future prospective studies integrating DCG features and involving larger, multicenter cohorts may further enhance the clinical applicability and robustness of the proposed radar framework in real-world settings.

## Conflicts of Interest

The authors declare that there are no conflicts of interest regarding publication of this paper.

## Author Contributions

Chun-Chih Chiu, Wen-Te Liu, and Jiunn-Horng Kang devised and designed the study; Chun-Chao Chen, Yu-Hsuan Ho, Yu-Wen Huang, Zong-Lin Tsai, and Rachel Chien extracted data; Ying-Ying Chen, Nai-Wen Chang, Hung-Wen Lu, and Yen-Ling Chen analysed and interpreted data; Chun-Chih Chiu wrote the first draft; Kang-Yun Lee, Arnab Majumdar, Ju-Chi Liu, and Cheng-Yu Tsai contributed to the development of subsequent versions and approved the final article; Cheng-Yu Tsai is the guarantor. All authors have approved the final version for publication and have agreed on the journal to which the article will be submitted. All authors take full responsibility for the integrity and accuracy of the work.

## Ethical Considerations

The protocol for this retrospective study was reviewed and approved by the Institutional Review Board at the Office of Human Research of Taipei Medical University (approval no: TMU-SHH: N202407022). Patient information, biochemical data, and relevant physiological variables were obtained through medical records. All procedures involving data retrieval, processing, analysis, and storage were conducted in accordance with officially approved protocols.

## Supplementary Materials

Supplementary Materials
