# Supplementary material for: Evaluating Cardiac Impairment From Abnormal Respiratory Patterns: Insights From a Wireless Radar and Deep Learning Study
Source: IEEE J Transl Eng Health Med. 2025 Jul 14;13:323–32. doi: 10.1109/JTEHM.2025.3588523 (PMC12310173; doi:10.1109/JTEHM.2025.3588523)
Supplement: Supplementary Materials [file supp1-3588523.docx]

**Supplementary Materials**

**Supplementary Figure S1. Architecture of Hybrid Deep Learning Models for Respiratory Event Prediction Using Radar-derived Signals.**

The established hybrid models combine deep neural networks with decision tree structures, namely deep neural decision trees. The model was configured with the following hyperparameters: a single decision tree, a learning rate of 0.05, a batch size of 400, 200 training epochs, an 11-layer neural network, and a feature usage rate of 0.5.


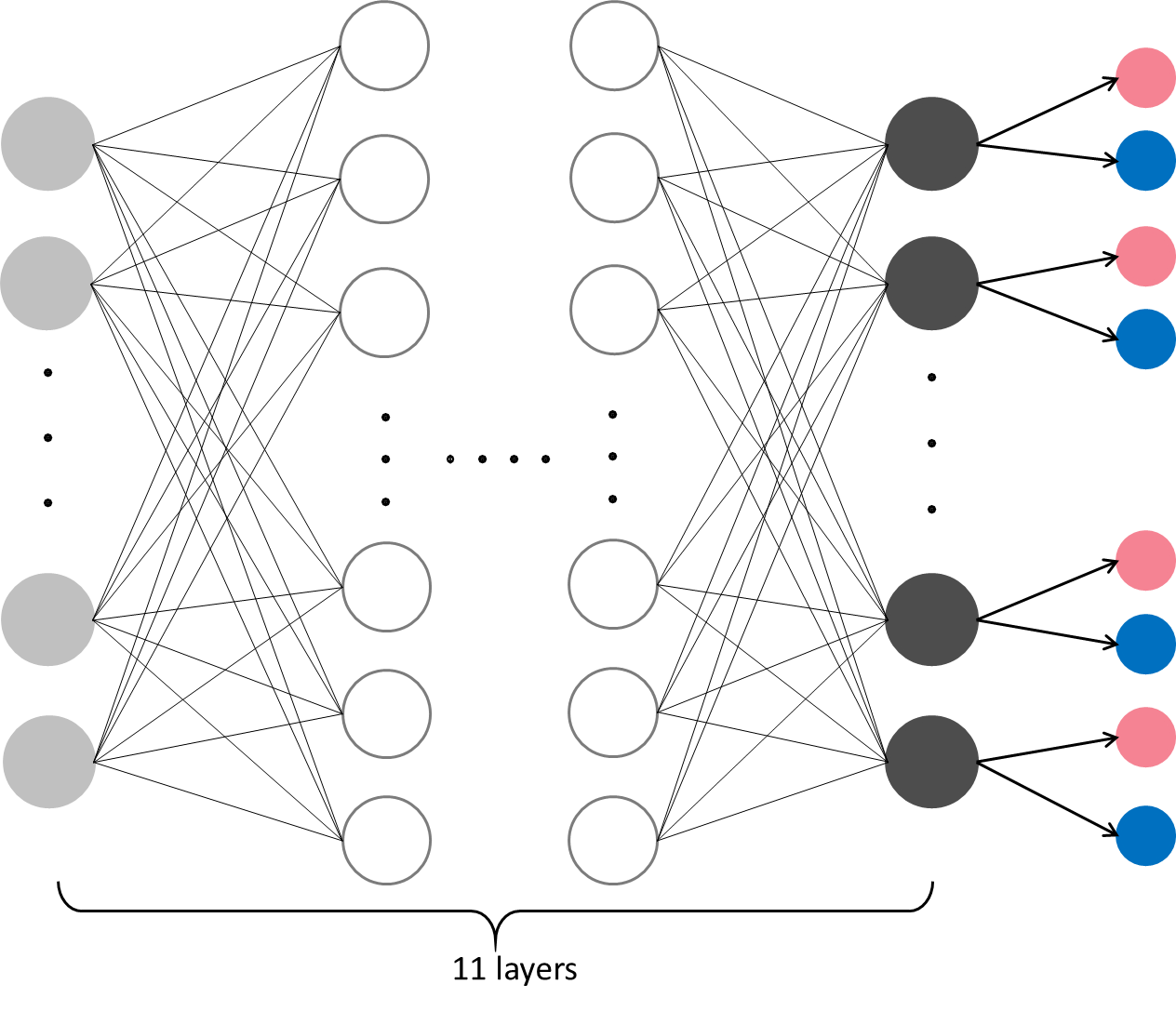


**Supplementary Table S1. Specifications of the Continuous-wave Radar Device**

| Parameter | Value |
| --- | --- |
| Waveform type | Continuous wave |
| Sensor size (cm) | 12 × 8 × 4 |
| Radio frequency (GHz) | 24 |
| Wavelength (mm) | 12.5 |
| Sampling frequency (Hz) | 50 |

**Supplementary Table S2. Comparison of the Proposed Continuous-wave Radar Framework with Other Commonly Used Approaches**

| Variable | Proposed continuous-wave radar | Frequency-modulated continuous-wave radar | Video-based | Wearable sensor |
| --- | --- | --- | --- | --- |
| System cost | US$120/month | US$200–300+ | Depends on camera type | Depends on devices and equipment |
| Multiple subject detection | Not supported | Yes | Yes | One sensor per person |
| Privacy protection | No imaging | No imaging | Captures face/body | No imaging |
| Target range (m) | 1–1.5 | 0.2–5+ | 0.3–2 | Requires attachment |
| Monitoring duration | Long-term | Long-term | Short-term | Short-term |
| Power consumption | Very low | Low to moderate | Moderate to high | Depends on sensor type |
| Setup complexity | Simple | Moderate | Moderate | Moderate |

**Supplementary Table S3.** **Performance of Established Hybrid Models for Predicting Respiratory Event Occurrence (i.e., apnea and hypopnea)**

| Variable | Value (%) |
| --- | --- |
| Accuracy | 75.78 |
| Sensitivity | 75.34 |
| Precision | 74.68 |
| Specificity | 76.19 |
| F1 Score | 75.01 |
| Note: the dataset included 15,264 apnea and hypopnea events, along with 14,232 segments of normal data with no identified events. | |
